# Effectiveness of a quality improvement curriculum for medical students

**DOI:** 10.3402/meo.v20.27133

**Published:** 2015-05-08

**Authors:** Kimberly M. Tartaglia, Curt Walker

**Affiliations:** Department of Internal Medicine, Wexner Medical Center, Ohio State University, Columbus, OH, USA

**Keywords:** medical education-systems-based practice, medical education-practice-based learning, quality improvement

## Abstract

**Introduction:**

As health systems find ways to improve quality of care, medical training programs are finding opportunities to prepare learners on principles of quality improvement (QI). The impact of QI curricula for medical students as measured by student learning is not well delineated. The aim of this study is to evaluate the effectiveness of a QI curriculum for senior medical students as measured by student knowledge and skills.

**Methods:**

This study was an observational study that involved a self-assessment and post-test Quality Improvement Knowledge Application Tool (QIKAT) for intervention and control students. A QI curriculum consisting of online modules, live discussions, independent readings and reflective writing, and participation in a mentored QI project was offered to fourth-year medical students completing an honor's elective (intervention group). Senior medical students who received the standard QI curriculum only were recruited as controls.

**Results:**

A total of 22 intervention students and 12 control students completed the self-assessment and QIKAT. At baseline, there was no difference between groups in self-reported prior exposure to QI principles. Students in the intervention group reported more comfort with their skills in QI overall and in 9 of the 12 domains (p<0.05). Additionally, intervention students performed better in each of the three case scenarios (p<0.01).

**Discussion:**

A brief QI curriculum for senior medical students results in improved comfort and knowledge with QI principles. The strengths of our curriculum include effective use of classroom time and faculty mentorship with reliance on pre-existing online modules and written resources. Additionally, the curriculum is easily expandable to larger groups of students and transferable to other institutions.

The Institute of Medicine reports *To Err is Human* and *Crossing the Quality Chasm* brought concepts of patient safety and healthcare quality to the forefront of medicine (1, 2). As healthcare delivery has become more complex, health systems are forced to adapt and find solutions to monitor patient safety and improve quality of care. In response to an increasingly complex process of healthcare delivery, medical training programs (medical schools, residencies, and fellowships) have sought opportunities to prepare learners for success in implementing safety measures and providing quality care through instruction of quality improvement (QI) principles. Review of QI instruction has primarily focused on curriculum development, highlighting didactic education, and experiential learning (3–7).

In addition to teaching QI principles, institutions have struggled with how to meaningfully assess their learners’ knowledge, skills, and attitudes related to QI. Examples of published experience include reporting on learner attitudes through surveys and identifying the types of projects and outcomes that resulted from the resident or student involvement in a quality project (8–10). Ogrinc et al. published the impact of a quality curriculum for internal medicine residents that measured learning using a 12-item self-assessment and Quality Improvement Knowledge Application Tool (QIKAT) (11). Using this tool, learners were given three scenarios and asked to propose an aim, measures, and potential interventions in response to the scenario. The use of the QIKAT as an assessment tool was replicated by Vinci et al., again used to assess the impact of a QI curriculum for Internal Medicine residents (5). Recently, the Pritzker School of Medicine published their experience with implementation of a quality and safety track (12). However, the evaluation of their curriculum was based only on student self-assessment of comfort with QI principles. To our knowledge, reports on the use of the knowledge portion of the QIKAT to evaluate QI curricula in medical students have not been published.

The aim of this study is to evaluate the effectiveness of a QI curriculum for senior medical students as measured by student knowledge and skills. We hypothesize that students who participate in our QI curriculum will have improved knowledge as measured by the QIKAT as well as a higher self-assessment of QI skills.

## Methods

### Participants and Setting

The participants in our study included fourth-year medical students who were applying to Internal Medicine residency programs and enrolled in an honor's elective in Internal Medicine. During that elective, students completed modules on QI, leadership, education, evidence-based medicine, and physical diagnosis. A convenience sample of fourth-year medical students applying to Internal Medicine residencies but who were not participating in the Honor's elective was recruited as controls. Control students received the standard curriculum in QI, which consisted of the Institute of Medicine reports, and an electronic module resource (60 min) that outlined the principles and methodology of QI.

### Curriculum Description

The conceptual framework for the QI curriculum was to provide students with basic knowledge and experience with QI principles to be prepared to enter residency as a fully engaged member of the healthcare team. Given the lack of background QI knowledge in traditional medical school curricula and the longitudinal nature of the elective, we needed to create a curriculum that allowed for significant portions to be completed by students asynchronously. The QI module embedded in the honor's elective spanned 9 months and included approximately 40 h of exposure time. The curriculum consisted of four parts: 1) Completion of the Institute for Healthcare Improvement (IHI) modules on QI, 2) Composition of a reflective writing based on their response to writings by Atul Gawande, 3) Discussion during two sessions with a content expert to reinforce and highlight important quality principles, and 4) Participation in a mentored QI project with a faculty member. Students enrolled in the Honor's elective completed these activities by assigned dates and presented their quality project to a general faculty audience at a spring reception. For their quality project, students worked individually or in pairs, and projects varied in setting (outpatient vs. inpatient) and specialty (medical vs. surgical) (Table 1).

**Table 1:** List of student projects during QI curriculum

2009–2010 Academic Year	Goal Directed Therapy for Severe Sepsis and Septic Shock
	Central Line Associated Blood Stream Infections in the Surgical ICU
	Antimicrobial Stewardship Program
	Reducing the Readmission Rates for Patients with Heart Failure with Normal Ejection Fraction
	Inpatient Asthma Guideline Compliance and Documented Best-Practices
2010–2011 Academic Year	Medication Reconciliation in the IM Residents’ Clinic
	Early Outpatient Follow-Up after Discharge for Heart Failure
	Improving Inpatient Hyperbilirubinemia Care
	Reducing Bleeding Risk in Patients on Coumadin
	Relearning the H&P
	DVT Prophylaxis Revisited
2011–2012 Academic Year	Patient Safety Rounds and Safety Culture
	Improving Patient Discharge Education by Medical Students
	Reducing Hospital-Acquired *C. difficile* Infections
	Improving Hyperbilirubinemia Management
	Best Practices: Stage 3 Chronic Kidney Disease
	Assessing Appropriate Left-Ventricular Assist Device (LVAD) Placement in Patients with Severe Systolic Heart Failure
	Feasibility of Antibiotic Time-Outs in the Inpatient Setting

### Study Design

The study was conducted over 3 years (2010–2013) as an observational study that involved a self-assessment and post-test (QIKAT) for intervention and control students. The QIKAT and self-assessment were administered in the spring of students’ fourth year, upon the completion of the QI curriculum for intervention students but prior to their spring presentation of their QI project. The self-assessment consists of 12 questions, and students were asked to rate their comfort on a four-point scale from 1 (Not at all comfortable) to 4 (Extremely Comfortable). The QIKAT consists of three scenarios (based in the emergency department, outpatient office, and dialysis unit of hospital) for which participants answered three questions related to the aim of a potential QI project as well as associated measures and potential interventions. Participant's responses were coded by one investigator (CW) and scored using a standardized scoring system by a blinded rater (KT). Each QIKAT scenario was scored 0–5 with a total possible score of 15 using the scoring system published by Vinci (Table 2) (5). Previous QI experience by participants was collected through self-report.

**Table 2:** Scoring system for QIKAT scenarios*

Category	Points
Aim	2 points for excellent aim, 1 point for good aim
Measure	1 point for good measure
Intervention	1 point for feasible intervention
Overall	1 point for all answers related
Total	5 points possible per scenario; 15 total possible points (3 scenarios)

* Published by University of Chicago Quality Assessment and Improvement Curriculum.

### Statistical analysis

All analyses were completed and study hypotheses tested using SPSS 21.0 (IBM Corp.). Statistical significance was defined as p<0.05. We performed frequency analyses to assess sample distribution and a series of chi-square tests of association to assess sample differences in sample sizes and prior exposure to QI between control and intervention groups. We performed a series of independent samples t-tests to test our study hypotheses. Finally, we calculated effect sizes for each outcome variable to assess the extent of difference between mean values in comfort with QI principles and case scenario evaluations.

## Results

Twenty-two students completed the QI curriculum embedded in an Honor's Internal Medicine elective and all students (100%) completed the self-assessment and QIKAT. Twelve students who were applying to Internal Medicine but who participated in only the standard QI curriculum completed the self-assessment and QIKAT as controls. Results of a chi-square test of association indicate no differences between control and intervention group sample sizes, χ^2^(2)=4.28, p=0.12. When examining differences in students’ exposure to QI, results of a series of chi-square tests of association suggest no differences between participants with prior exposure and those without in both study groups, control (χ^2^(2)=1.71, p=0.42) and intervention (χ^2^(2)=3.85, p=0.15). Results suggest sample sizes in the control and intervention groups are adequately similar to combine across classes for all study analyses.

Students who completed the QI curriculum reported greater comfort with their skills in QI principles than students who completed the standard curriculum. Intervention students had a higher mean value of comfort with QI principles using a composite measure which combined the scores of all 12 domains; t(32)=−4.88, p<0.01 (95% CI: −0.99 to −0.41). Students who completed the QI curriculum also reported increased comfort in nine of the 12 domains (p<0.01) (Table 3). There were no differences between intervention and control students noted in the ability to write a clear problem statement, use measurement to improve skills, or implement a structured plan to test a change.

**Table 3:** Self-assessed comfort with quality improvement principles

Quality improvement principle	t-test	Control mean^a^ (SD)	Intervention mean^b^ (SD)
1. Writing a clear problem statement (goal, aim)	t(32)=−1.53	**3.00** (0.60)	**3.32** (0.57)
2. Applying the best professional knowledge best professional	t(32)=−3.43*	**2.58** (0.51)	**3.23** (0.53)
3. Using measurement to improve your skills	t(32)=−2.00	**2.83** (0.72)	**3.27** (0.55)
4. Studying the process	t(32)=−3.06*	**2.50** (0.67)	**3.32** (0.78)
5. Making changes in a system	t(32)=−3.10*	**2.00** (0.74)	**2.82** (0.73)
6. Identifying whether a change leads to an improvement in your skills	t(32)=−3.76*	**2.67** (0.49)	**3.32** (0.48)
7. Using small cycles of change	t(32)=−2.64*	**2.58** (0.51)	**3.23** (0.75)
8. Identifying best practices and comparing these to your local practice	t(32)=−3.87*†	**2.75** (0.45)	**3.45** (0.60)
9. Implementing a structured plan to test a change	t(32)=−1.60	**2.58** (0.67)	**3.00** (0.76)
10. Using the PDSA model as a systematic framework for trial and learning	t(32)=−6.16*	**1.58** (0.76)	**3.32** (0.78)
11. Identifying how data is linked to specific processes	t(32)=−2.30*†	**2.50** (1.00)	**3.23** (0.61)
12. Building your next improvement upon prior success or failure	t(32)=−2.34*	**2.83** (0.58)	**3.36** (0.66)

a n=12

b n=22

* p<0.01

† Equal variances not assumed.

Students who completed the QI curriculum also performed better on the series of case scenarios than students who did not complete the curriculum. Results of an independent samples t-test indicated a difference in the mean value of the composite score of the case scenarios (p<0.01, 95% CI: −7.04 to −4.08). Furthermore, we noted statistically significant differences between intervention and control groups on all three case scenarios (Table 4).

**Table 4:** Results of QIKAT case scenarios

Case scenarios	t-test	Control mean^a^ (SD)	Intervention mean^b^ (SD)
1. Case 1	t(32)= −7.42*	**1.67** (0.89)	**3.95** (0.84)
2. Case 2	t(32)= −3.91*	**2.50** (0.90)	**3.82** (0.96)
3. Case 3	t(32)= −6.73*	**2.00** (0.85)	**3.95** (0.78)
4. Composite	t(32)= −7.63*	**6.17** (1.95)	**11.73** (2.07)

a n = 12

b n = 22

* p < 0.01.

We calculated the effect sizes for each outcome variable to determine the size of the observed difference between the mean values of control and intervention group scores, indicating the effectiveness of the intervention curriculum. Using the sample mean values and pooled standard deviation, we calculated effect sizes of Cohen's d=1.76 and d=2.74 (α=0.05; two-tailed test) for the outcome measures, comfort with QI principles and case scenario evaluations, respectively. The observed differences were large (d>0.80), according to Cohen's conventions (13).

## Discussion

Our results demonstrate an association between a succinct QI curriculum for senior medical students and improved comfort and knowledge with QI principles compared with control students. The QIKAT scores for students in our curriculum were favorable compared to other studies. Our intervention composite score was 11.7 which is similar to the post-test mean reported by Ogrinc (11.4) and slightly higher than the post-test mean reported by Vinci 9.7 (5, 11). Additionally, student self-assessed comfort with QI principles in both the control and intervention groups in our study were similar to scores reported by Ogrinc (11).

The strength of our curriculum is the effective use of independent, asynchronous resources in addition to high-yield time with a content expert. Our use of IHI modules and writings by professionals in quality and patient safety allow the student to complete much of the curriculum at a time that is convenient for them. Additionally, students are able to successfully apply their QI knowledge by working alongside a faculty member to complete a QI project. Student comments during the end-of-course evaluation suggest that for many, the QI module and associated project work was the highlight of their Honor's elective experience (Box 1).

Box 1 **Representative student comments for QI curriculum**

‘This was my favorite part of the course. I received the appropriate amount of guidance throughout the project. I learned a great deal and felt like I really contributed to the community’.

‘This was one of the most effective modules. The IHI open school was actually very helpful’.

‘The QI module is the best part of the course. It is a very helpful and applicable module for next year’.

‘I really enjoyed the QI part of the honors elective. I would like to hopefully pursue some QI during residency now, and feel like I have the tools to do it’.

‘My favorite module of the Honors IM course. The skill set and vocabulary I gained already helps me communicate with leadership in the medical community’.

Limitations of this study include a single site with a small sample size and lack of pre/post-design. Our sample size was limited by the size of the Honor's IM elective which enrolled six to eight students per year. Our decision to pursue a post-test only design was based on the concern that participants would learn from the pretest (14). Furthermore, although members of the intervention and control were drawn from the same population, participation in the intervention was voluntary and resulting scores could be attributed to characteristics we did not measure prior to assignment. Significant differences in mean values and magnitude of effect sizes in the evaluations between control and intervention groups does, however, suggest that exposure to this QI curriculum relates to increased knowledge of and comfort with QI principles. Additionally, we do not have data on the downstream impact of this curriculum (i.e., whether this curriculum impacted students’ performance on QI initiatives in residency).

This curriculum is easily transferable to other institutions and expandable to larger groups of students. We have shared our curriculum framework with three institutions and have now rolled this curriculum out to all medical students in the OSU College of Medicine *Lead.Serve.Inspire (LSI)* curriculum (started August 2012). In the *LSI* curriculum, students complete the IHI modules and foundational sessions with a content expert during the first 18 months before participating in rapid-cycle improvement projects during their clinical immersions. The students work in small groups to complete these projects which are facilitated by a faculty expert and institutional quality manager. Future directions include monitoring learning outcomes for students in the *Lead.Serve.Inspire* curriculum and obtaining post-graduation information for students who have completed the Honor's IM elective.
